# Supplementary material for: Inter-limb asymmetry in youth elite soccer players: Effect of loading conditions
Source: PLoS One. 2022 Jun 24;17(6):e0269695. doi: 10.1371/journal.pone.0269695 (PMC9231726; doi:10.1371/journal.pone.0269695)
Supplement: Attachment — Submitted filename: Response to Reviewers .docx [file pone.0269695.s002.docx]

Dear Editor,

My co-authors and I would be grateful for you to consider our **revised** manuscript, “**Inter-limb asymmetry in youth elite soccer players: effect of loading conditions**”, for potential publication as an original investigation in *Plos One*.

Below, I have indicated the applied edits in the manuscript file using red highlighted text.

Giampiero Ascenzi, MSc

Aspire Academy & Qatar FA

PO Box 22287, Doha, Qatar

e-mail: giampiero.ascenzi@hotmail.it

5. Review Comments to the Author

**Reviewer #1**: Abstract

“rear-foot-elevated-split-squat-test (body mass, body mass 25% body mass 50%)”. Reading the following part of the article, “body mass 25%” mean “25% of the body mass”. If this interpretation is correct, it could be clearer reported.

**We thank the Reviewer for the observation. We clarified this point in our revised manuscript.**

Participants

Some information about the nationality of the observed players could be reported to better define the experimental sample, because players’ competitive level could also depend by the national context.

**Thank you for this comment. We have now provided the relevant details as per the Reviewer’s suggestion.**

Results

Table 1 and 2 should be better structured and readable, by reporting rows which are able to define the subareas of results.

**Thank you for this comment. We have attended to this suggestion.**

S3 and S4 Figs could be better visible (higher definition)

**Thank you for this comment. The poor image definition is likely due to issues with the Editorial Manager system.**

Discussion

Line 193. Regardless of the number of participants, repeated measures on the same trials, along different phase of a season, and controlling the players internal training load, could enlarge the point of view on the magnitude and the direction of the inter-limb asymmetry using the same test with different loads.

**Thank you for this comment. However, we are not sure about the relevance of the Reviewer’s comment. Specifically, we conducted a one-off assessment that, similar to a typical maximal oxygen uptake assessment, is practically independent from routine monitoring of players over time. Therefore, reporting measures of internal load is out of context given the underlying purpose of our study.**

**Reviewer #2**: L44: Full stop mark is missing just after the reference.

**Thank you for this comment. We have attended to this suggestion.**

L55-56: This section requires a more in-depth explanation. Why rear-foot-elevated-split-squat-test (RFESS) can be regarded as a practical method for measuring unilateral strength in sport?

**We thank the Reviewer for the observation. We informed our study design and procedure given previous studies adopting the assessment having practical relevant to us.**

L69: "IKF" and "IKE" acronyms are reported here for the first time. Please explain the meaning within the text.

**Thank you for this comment. We have attended to this suggestion.**

L92-93: What was the range of movement of knee flexion and how did the authors assessed it during the RFESS? Please report this information.

**Thank you for this comment. We have now provided the relevant details in our Methods section.**

***A professional goniometer (Baseline® Measurement, White Plains, NY, USA) was used to assess the 90° knee angle which started the maximal ballistic push off (positive phase, upward extension).***

L98: Warm up is the same on Day 2 as on Day 1? Please report this information.

**Thank you for this comment. We have now provided the relevant details in our Methods section.**

L100: "DL" and "NDL" acronyms are reported here for the first time. Please explain the meaning within the text.

**Thank you for this comment. We have now clarified this point in our Methods section.**

L100-103: Please report the references to support the choice of the angular velocities that have been used for the isokinetic test.

**We have attended to the Reviewer’s suggestion and cited the relevant source for the angular velocities we selected for our isokinetic assessments.**

L103-104: Please report the hip angle or if in an upright position, the backrest angle.

**Thank you for this comment. We have provided the relevant details at line 119-120 of our revised manuscript.**

L115-116: Did the authors assumed normal distribution or did they verify it in order to chose the most suitable statistical hypothesis test? Please report this information.

**Thank you for highlighting such an important point relevant to our statistical analysis section, which we have clarified.**

***Visual inspection of frequency distributions of the raw strength assessment data suggested assumptions of normality were not violated.***

L136: Data in Table 2 look to be inconsistent. Values of Dominant and Non-dominant leg are reported in column 2 and 3 but at L72 the asymmetry was calculated between stronger and weaker limb. Is always dominant leg the stronger and the non-dominant the weaker?

**We thank the Reviewer for the important observation since it represented an inconsistency of our original submission. We have provided the relevant details for the formula that applies to our study.**

L163-164: Reference is required for this statement.

**We have attended to the Reviewer’s suggestion and cited the relevant source.**

L151: Discussion section should be largely improved. For instance, the aim of this study is "...to evaluate the relationship of the magnitude and the direction of inter-limb asymmetries measured with different strength testing modes and/loads in a sample of elite youth soccer players". The 1) magnitude is evaluated by the inter-limb asymmetries formula at L72 but it is not clear how do you evaluate 2) the "direction of the inter-limb asymmetries" and 3) the relation between 1) and 2). Please clarify this point.

**Thank you for the sensible observations and suggestions regarding our Discussion section. We attempted to address the Reviewer’s comment by expanding relevant parts of our Discussion in our revised manuscript.**

L174-179: This paragraph does not make much sense and it should be rewritten. It seems more review of the literature about the available methods to assess strength asymmetries and imbalances rather than fully expressing the “whys” of this study. Authors should also focus on better explaining their results by discussing the task-specificity of inter-limb asymmetries and the need that more than a single test should be used to profile muscular imbalances (e.g., Bishop C, Lake J, Loturco I, Papadopoulos K, Turner A, Read P. Interlimb asymmetries: The need for an individual approach to data analysis. J Strength Cond Res, 2018; (May): 1–7).

**Thank you for this comment. We have now modified this paragraph according to the suggestion of the revisor.**

L179, 182: Full stop mark is missing just after the reference.

**Thank you for this comment. We have now included the full stop marks after the refences.**

L188: This sentence should be rephrased by avoiding negations (e.g., Nevertheless, limitations in our study are...)

**Thank you for this comment. We have attended to this suggestion.**

6. We note that Figure 1 and 2 includes an image of a participant. 
As per the PLOS ONE policy (http://journals.plos.org/plosone/s/submission-guidelines#loc-human-subjects-research) on papers that include identifying, or potentially identifying, information, the individual(s) or parent(s)/guardian(s) must be informed of the terms of the PLOS open-access (CC-BY) license and provide specific permission for publication of these details under the terms of this license. Please download the Consent Form for Publication in a PLOS Journal (http://journals.plos.org/plosone/s/file?id=8ce6/plos-consent-form-english.pdf). The signed consent form should not be submitted with the manuscript, but should be securely filed in the individual's case notes. Please amend the methods section and ethics statement of the manuscript to explicitly state that the patient/participant has provided consent for publication: “The individual in this manuscript has given written informed consent (as outlined in PLOS consent form) to publish these case details”.

**Thanks to the journal editors to highlight this point. Please note that the images are not from any participants of the study but is an exemplar by the first author (Giampiero Ascenzi).**

We note that your Data Availability Statement reads: "All Supporting files are available from the excel database attached to the manuscript."

Please review the journal's policy on identifying information here: https://journals.plos.org/plosone/s/data-availability#loc-acceptable-data-access-restrictions

Data sharing should never compromise participant privacy. It is therefore not appropriate to publicly share personally identifiable data on human research participants. The following are examples of data that should not be shared:

Name, initials, physical address

Internet protocol (IP) address

Specific dates (birth dates, death dates, examination dates, etc.)

Contact information such as phone number or email address

Location data

Data that are not directly identifying may also be inappropriate to share, as in combination they can become identifying. For example, data collected from a small group of participants, vulnerable populations, or private groups should not be shared if they involve indirect identifiers (such as sex, ethnicity, location, etc.) that may risk the identification of study participants.

As your dataset contains identifying information in the form of exact dates (both birthdays and the days of data collection) and ages more specific than integer years, please remove these values and reupload your fully anonymized dataset at this time. To anonymize the ages, for example, please remove all decimal points (i.e. 42.4 years -> 42 years).

**Thanks to the journal editors to highlight this point. I have deleted all the information relating to the date of birth and the day of the tests. In addition I have removed the decimal number from the age**
