# Supplementary material for: Inter-limb asymmetry in youth elite soccer players: Effect of loading conditions
Source: PLoS One. 2022 Jun 24;17(6):e0269695. doi: 10.1371/journal.pone.0269695 (PMC9231726; doi:10.1371/journal.pone.0269695)
Supplement: Attachment — Submitted filename: Response to Reviewers.docx [file pone.0269695.s003.docx]

Dear Editor,

My co-authors and I would be grateful for you to consider our **revised** manuscript, “**Inter-limb asymmetry in youth elite soccer players: effect of loading conditions**”, for potential publication as an original investigation in *Plos One*.

Below, I have indicated the applied edits in the manuscript file using red highlighted text.

Giampiero Ascenzi, MSc

Aspire Academy & Qatar FA

PO Box 22287, Doha, Qatar

e-mail: giampiero.ascenzi@hotmail.it

**Review Comments to the Author**

Please use the space provided to explain your answers to the questions above. You may also include additional comments for the author, including concerns about dual publication, research ethics, or publication ethics. (Please upload your review as an attachment if it exceeds 20,000 characters).

**Reviewer #1:**

(No Response)

**Reviewer #2:**

Dear authors,

Your latest review has improved the quality of the manuscript. Please check for consistency when you address to "Non-dominant" within the text. @L120 and Tab1 you report "No-dominant" instead of "Non-dominant". Please check.

**Thank you for this comment. We have attended to this suggestion concerning the use of abbreviations in our manuscript.**
